# Dual Role of MSC-Derived Exosomes in Tumor Development

**DOI:** 10.1155/2020/8844730

**Published:** 2020-09-09

**Authors:** Rou Zhao, Xinke Chen, Hui Song, Qingli Bie, Bin Zhang

**Affiliations:** ^1^Department of Laboratory Medicine, Affiliated Hospital of Jining Medical University, Jining, Shandong, China; ^2^Department of Microbiology, Qingdao University Life Science College, Qingdao, Shandong, China; ^3^Institute of Forensic Medicine and Laboratory Medicine, Jining Medical University, Jining, Shandong, China

## Abstract

Mesenchymal stem cells (MSCs) are a class of adult stem cells derived from the mesoderm. They can self-renew, have multidirectional differentiation potential, and can differentiate into a variety of mesenchymal tissues. MSCs can produce a large number of exosomes, which can mediate information exchange and transmission between cells in the tumor microenvironment under conditions of rest or stress. Recent studies have reported conflicting findings regarding the effect of MSC-derived exosomes on tumors. Some studies have suggested that MSC-derived exosomes can promote tumor growth and metastasis, but others have reported that they can inhibit tumor cell growth. Here, we investigate the two sides of the debate regarding the effect of MSC-derived exosomes on tumors and analyze the reasons for the divergent findings.

## 1. Background

Mesenchymal stem cell- (MSC-) derived exosomes were first studied in a mouse model of myocardial ischemia/reperfusion injury in 2010 [1]. Under transmission electron microscopy, MSC-derived exosomes exhibit characteristic circular shapes of varying sizes [2]. Cross-talk between the tumor microenvironment and tumor seems to be crucial for tumor growth and development. Related studies have shown that MSCs produce exosomes, which may act as paracrine mediators by transferring signal molecules that regulate tumor cell proliferation, angiogenesis, and metastasis by controlling many cellular pathways. At present, whether MSC-derived exosomes promote or inhibit tumors remains controversial, and many studies have reported inconsistent conclusions. As listed in Tables 1 and 2, emerging evidence indicates that MSC exosomes may transfer proteins, messenger RNA, and microRNA to recipient cells, thereby affecting tumor cell growth, metastasis, and drug response. Here, we review the relationships identified in recent years between MSC-derived exosomes and tumor development, with an emphasis on how MSC-derived exosomes appear to play two contrasting roles, and discuss the mechanisms of these roles.

## 2. Characteristics of MSC-Derived Exosomes

MSCs have attracted considerable attention in recent years because of their capacities for immunoregulation and tissue repair and their implications for tumor development. MSCs are a class of mesoderm pluripotent stem cells, with pluripotent differentiation potential [3, 4], which are preferentially present in the niches of the perivascular spaces of almost all human tissues and organs, including the dental pulp [5], bone marrow [6], adipose tissue [7], neonatal placenta [8], amniotic membrane, and umbilical cord [9]. Under examination with an inverted microscope, MSCs appear as relatively uniform fibroblast-like cells with rotational filling adherent growth [10]. The characteristics of MSCs change depending on the pathophysiological state of the tissue in which they are located [11]. Complex interactions between MSCs and molecules in the surrounding tissue microenvironment lead to various results, depending on the type and duration of tissue damage and the intensity of the associated local inflammation [8–12]. Receptors for multiple factors are expressed on the surface of MSCs, which can effectively migrate to the site of inflammation or cancer because of the inflammatory factors in the microenvironment of such sites [13]. MSCs secrete a variety of inflammatory factors, such as MCP-1, IL-6, and IL-8, which contribute to the directed migration of MSCs [14]. Moreover, they have been found to migrate to tumors and evolve into tumor-associated MSCs (TA-MSCs) and cancer-associated fibroblasts (CAFs) [15, 16], which secrete a plethora of growth factors, cytokines, chemokines, and structural protein components to communicate with tumor cells and promote tumor development by activating cell proliferation and invasion, angiogenesis, and other processes [17]. Recent studies involving the use of MSCs as cell carriers in targeted tumor therapy have shown that genetically modified MSCs can continuously and stably produce therapeutic factors that play roles in tumor inhibition after reaching inflammation or tumor sites [18]. Current experimental models have indicated that MSCs may promote or inhibit the regulation of various tumors; however, the mechanism by which MSCs regulate tumor cells remains unclear.

**Figure 1:**
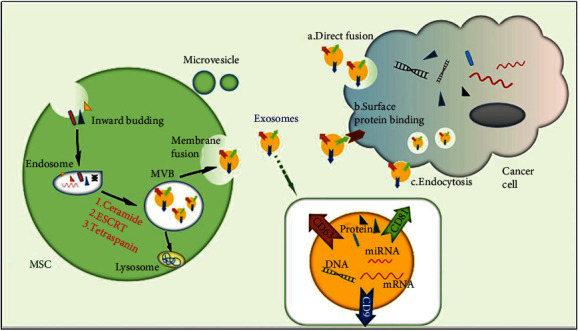
Exosome biogenesis and secretion. Cell membrane inward budding leads to the formation of early endosomes, and these transform into multivesicular bodies (MVBs) with a dynamic subcellular structure. MVBs can be fused with lysosomes or the plasma membrane and secreted exosomes. Finally, exosomes release cargo such as DNA, microRNA, and proteins to cancer cells through (a) direct fusion with recipient cell plasma, (b) surface protein binding, or (c) endocytosis.

Exosomes are small membrane vesicles that contain complex RNA and proteins. They are typically discoid vesicles 30–100 nm in diameter and were first identified in sheep reticulocytes in 1983 [19]. Exosomes are secreted by a variety of cells, including endothelial cells, immune cells, platelets, and smooth muscle cells. Exosomes are mainly derived from the intracellular polyvesicles formed by intracellular lysosomal particles, which are released into the extracellular matrix by the fusion of the extracorporeal membrane of polyvesicles with the cell membrane. When secreted into receptor cells by host cells, exosomes regulate the biological activities of receptor cells by carrying proteins, nucleic acids, lipids, and other molecules that have effects on receptor cells [20, 21]. Under the condition of physiological or pathological changes, exosomes can mediate intercellular communication and regulate the biological activities of recipient cells through their specific cargo [22]. The formation of exosomes begins with an inward budding of the cell membrane, resulting in the formation of early endosomes that incorporate membrane protein; they then transform into multivesicular bodies (MVBs) with a dynamic subcellular structure [23]. As shown in Figure 1, MVBs can be generated through two mechanisms: endosomal sorting complex required for transport (ESCRT) and independent ESCRT [24]. The ESCRT mechanism is activated by a set of cytoplasmic protein complexes that recognize ubiquitin-modified membrane proteins. Ubiquitin markers identified by the first ESCRT compound, ESCRT 0, enrich the inner body membrane and pass the ubiquitinated substances to ESCRT I and ESCRT II. Tsg101 in ESCRT I recognizes disulfide bonds and induces endosomal membrane depression; bonds are subsequently cut by ESCRT I to form MVBs [25]. However, MVBs can be formed without ESCRT. For example, the accessory protein, alg-2 interacting protein X, can directly bind with intracellular adaptor proteins to participate in the formation of exosomes. The production of these ESCRT-independent MVBs can promote MVB formation through tetraspanins [26] and ceramide-induced cell membrane budding [27]. MVBs can be fused with lysosomes that have degraded and recycled content and can also be fused with the plasma membrane and secreted outside of the cell. Exosomes are vital for intercellular communication and play a major role in paracrine. Exosomes can directly bind to specific ligands of target cells through surface receptors, triggering intracellular signal transduction or transferring exome surface receptors to target cells [28]. Multimolecular complexes can also be formed on the surface of target cell membranes to change the structure of the lipid bilayer and directly endorse exosomes, or a mass of membrane fusion proteins in the target cell membrane and exosome can overcome the energy barriers of plasma membrane fusion and achieve membrane fusion [29]. Exosomes can be separated through differential centrifugation, use of an ExoQuick exosome extraction kit, immunomagnetic bead sorting, sucrose density gradient ultracentrifugation, microfluidic separation, and other methods [30, 31].

## 3. The Mechanism by Which MSC-Derived Exosomes Promote Tumor Progression

### 3.1. The Role of MSC-Derived Exosomal MicroRNA

**Figure 2:**
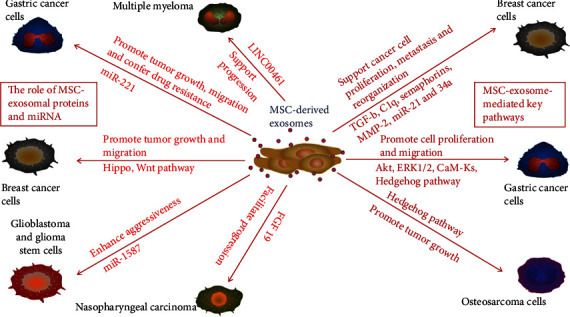
Promotional roles of MSC-derived exosomes in tumors. MSC-derived exosomes can promote tumor growth and cell migration and confer drug resistance on gastric cancer by activating the Akt, ERK1/2, CaM-Ks, and Hedgehog pathways and transporting miR-221 to cancer cells. MSC-derived exosomes support multiple myeloma progression by shuttling LINC00461. Moreover, MSC-derived exosomes support breast cancer cell proliferation, metastasis, and reorganization through Hippo and Wnt pathways and by delivering exosomal TGF-b, C1q, semaphorins, MMP-2, miR-21, and miR-34a. MSC-derived exosomes promote osteosarcoma growth through the Hedgehog pathway. MSC-derived exosomes facilitate nasopharyngeal carcinoma progression through the delivery of FGF19. MSC-derived exosomes enhance the aggressiveness of glioblastoma and growth of glioma stem cells through the delivery of miR-1587.

As shown in Figure 2, MSC-derived exosomes can promote tumor growth through a variety of mechanisms. Valadi et al. first found that exosomes contain both mRNA and microRNA, which can be delivered to another cell and be functional in the new location, and proposed that this RNA be called “exosomal RNA” [32]. Exosomes containing microRNAs are thought to regulate communication between stem cells and cancer cells [33]. Several recent studies have shown that exosomal miRNAs secreted by stem cells affect the biological behavior of cancer cells [34]. To analyze the effects of MSC-derived exosomal microRNAs on cancer cells, Figueroa et al. [35] analyzed the exosomal content and identified miR-1587 as a mediator of the exosomal-promoted effects of glioma-associated human MSCs (hMSCs) on glioma cells. Vallabhaneni and collaborators observed that exosomes released by serum-derived hMSCs could induce breast cell proliferation by transferring miRNA-21 and miR-34a [36]. In a study by Wang et al. [37], miR-221 was identified as a highly specific microRNA in exosomes from gastric cancer tissue-derived MSCs; the exosomes mediated the transfer of functional miR-221 to gastric cancer cells and promoted their proliferation and migration. However, Roccaro et al. [38] found that exosomal microRNA content differed between normal bone marrow-derived MSCs (BM-MSCs) and MM BM-MSCs; because of their relatively high content of the tumor suppressor miR-15a, exosomes derived from MM BM-MSCs promoted MM tumor growth, but normal BM-MSC exosomes inhibited the growth of MM cells. In recent years, multiple long noncoding RNAs (lncRNAs) have been involved in the regulation of MM development. In a study by Deng et al. [39], LINC00461 was transmitted by MSC-derived exosomes to multiple myeloma cells and enhanced cell proliferation and suppressed apoptosis by modulating microRNA/BCL-2 expression.

### 3.2. Role of MSC-Derived Exosomal Proteins

Proteins are one of the major components of exosomes [40]. Protein analysis of MSC-derived exosomes has demonstrated the presence of MMP-2 and MSC-specific markers, including CD90 and ecto-5′-nucleotidase. Yang et al. found that MSC-derived exosomes containing MMP-2 enzymes can alter cell function and recombine the cancer microenvironment, which is a new approach for improving cancer cell growth [41]. Because it is associated with poor outcomes of cancer, FGF19 may serve as a therapeutic target for treating cancer. FGF19 activity is regulated by the binding and activation of FGFR4, and this FGF19-FGFR4 interaction plays a role in carcinogenesis [42]. FGF19 was found to be highly expressed in MSC-derived exosomes, and exosomes stimulate NPC progression by activating the FGF19-FGFR4-dependent ERK signaling cascade [43]. Furthermore, Biswas and collaborators determined that MSC-derived exosomes but not exosomes from tumor cells had high levels of TGF-b and C1q, which enhance the immunosuppressive activity and M2 polarization of myeloid cells [44]. Our research team found that umbilical cord MSC-derived exosomes promote cell proliferation by transporting Wnt4 [45, 46].

### 3.3. Key Pathways Mediated by MSC-Derived Exosomes

Regardless of the type of molecule transported by MSC-derived exosomes, important pathways of target cells are activated by such transportation. In recent years, the effect of MSC-derived exosomes on tumor progression has been extensively studied. Among the proposed mechanisms, pathway activation mediated by MSC-derived exosomes has been widely investigated. Akt is one of the main downstream effects of PI3K and activates multiple signal phosphorylation substrates that significantly affect tumor cell growth and cell cycle progression. Gu et al. demonstrated that MSC-derived exosomes induce Akt phosphorylation, thereby enhancing the epithelial-mesenchymal transition (EMT) and self-renewal capacity of gastric cancer cells [47]. It has been reported that human bone marrow MSCs exist in the tumor microenvironment, participate in the formation of the tumor microenvironment, and interact with cancer cells [48]. To understand the mechanism of human bone marrow MSC- (hBMSC-) derived exosomes on tumor growth, Qi et al. [49] examined the signaling pathway of exosomes derived from MSCs isolated from human bone marrow tissue and found that hBMSC-derived exosomes promoted MG63 and SGC7901 cell growth through the activation of the Hedgehog signaling pathway. It was also demonstrated that hBMSC-derived exosomes enhance vascular endothelial growth factor (VEGF) expression in gastric carcinoma cells by activating the extracellular signal-regulated kinase 1/2 (ERK1/2) pathway [50]. Adipocytes are the most abundant stromal cell component in breast cancer tissues, and Wang et al. [51] used exosomes isolated from in vitro MSC-differentiated adipocytes to systematically investigate the effects of adipocyte exosomes on tumor development in a breast cancer model. They discovered that MSC-derived exosomes enhanced breast cancer cell proliferation and migration and protected breast cancer cells from serum derivation or chemotherapeutic drug-induced apoptosis through activation of the Hippo signaling pathway in vitro. In addition, exosomes were found to contribute to in vivo tumor growth in a mouse xenograft model. Exosomes from human adipose-derived MSCs were demonstrated to promote migration through the Wnt signaling pathway in a breast cancer cell model [52]. Growing evidence suggests that MSCs protect tumor cells from chemotherapeutic drugs by generating multiple factors, recycling macromolecules, and activating certain signal cascades. Ji et al. [53] found that MSC-derived exosomes induce drug resistance in gastric cancer cells by activating the CaM-Ks/Raf/MEK/ERK pathway.

## 4. Antitumor Effect of MSC-Derived Exosomes

### 4.1. Direct Antitumor Effects of MSC-Derived Exosomes

**Figure 3:**
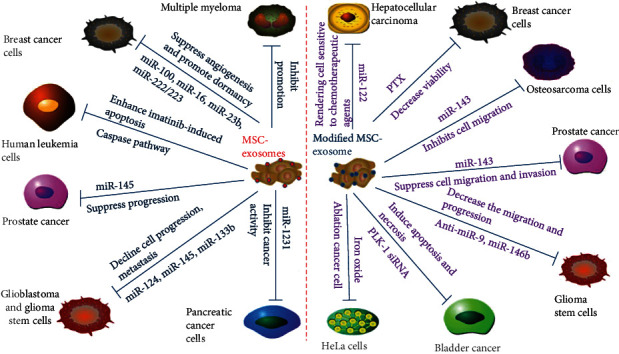
Inhibitory roles of MSC-derived exosomes in tumors. MSC-derived exosomes suppress angiogenesis and promote dormancy of breast cancer by shuttling miR-100, miR-16, miR-23b, and miR-222/223. MSC-derived exosomes suppress cell migration, invasion, and progression by shuttling miR-143 and miR-145. MSC-derived exosomes induce bladder cancer cell apoptosis and necrosis by transporting PLK-1 siRNA and inhibit osteosarcoma cell migration by shuttling miR-143. MSC-derived exosomes render hepatocellular carcinoma cells sensitive to chemotherapeutic agents through delivery of miR-122. MSC-derived exosomes enhance human leukemia cell imatinib-induced apoptosis through the caspase pathway. MSC-derived exosomes hinder the progression of glioblastoma and the progression and metastasis of glioma stem cells by transporting miR-124, miR-145, anti-miR-9, and miR-146b. MSC-derived exosomes inhibit pancreatic cancer activity and suppress glioma progression by shuttling miR-1231 and miR-133b. MSC-derived exosomes reduce breast cancer cell viability and the ablation of HeLa cells by transporting PTX and iron oxide.

Although most studies concerning MSC-derived exosomes have focused on their role in promoting tumor progression, a considerable body of other research has demonstrated that MSC-derived exosomes have significant antitumor effects (Figure 3). Takahara and collaborators indicated that microRNA-145 mediates the inhibitory effect of adipose-derived stem cells on androgen-independent prostate cancer [54]. Lee et al. demonstrated that the miR-124 and miR-145 delivered by MSC-derived exosomes significantly reduce the migration of glioma cells and the self-renewal of glioma stem cells [55]. Moreover, Xu et al. discovered that MSC-derived exosomes shuttle microRNA-133b to inhibit glioma progression through the Wnt/*β*-catenin signaling pathway by targeting EZH2 [56]. In addition, MSC-derived exosomes also significantly downregulate the expression of VEGF in breast cancer cells in vitro and in vivo. Pakravan et al. [57] hypothesized that the exosomal transfer of miRNAs from MSCs may affect tumor angiogenesis. Their findings suggest that MSC exosomal transfer of miR-100 suppresses in vitro angiogenesis through modulation of the mTOR/HIF-1*α*/VEGF signaling axis in breast cancer cells. This hypothesis was confirmed by another group. Lee et al. [58] demonstrated that MSC-derived exosomes significantly downregulate the expression of VEGF in breast cancer cells by transferring antiangiogenic molecule miR-16. Dormant breast cancer metastasizes into the bone marrow after prolonged dormancy and interacts with MSCs in the bone marrow. Ono et al. [59] reported that exosomes from bone marrow MSCs contain miR-23b, which promotes dormancy in metastatic breast cancer cells. Similarly, exosomal miR-222/223 was confirmed to stimulate cycling quiescence and early breast cancer dormancy in the bone marrow [60]. In addition, Shang et al. found that MSC-derived exosomal miRNA-1231 inhibits the activity of pancreatic cancer [61], and Liu et al. [62] demonstrated that MSC-derived exosomes enhance imatinib-induced apoptosis in human leukemia cells through activation of the caspase signaling pathway.

### 4.2. Antitumor Effects of Modified MSC-Derived Exosomes

Because of their membrane structure, exosomes have potential to serve as natural carriers of therapeutic agents for cancer therapy. Recent research has confirmed that modified exosomes enhance the cancer-killing efficacy and cancer-targeting ability of drugs, thereby increasing the effectiveness of individual cancer therapies [63]. Glioblastoma (GBM) is the most aggressive and common type of primary brain tumor, has extremely poor prognosis, and is highly resistant to conventional chemotherapy [64, 65]. The application of miR-targeting therapeutics in GBM treatment is an area of extensive research. Munoz et al. [66] demonstrated that the delivery of anti-miR-9 by MSC-derived exosomes to a GBM could reduce miR-9 expression and the resistance of GBM to TMZ. Katakowski et al. transfected MSCs with the miR-146b expression plasmid and reported that intratumor injection of exosomes derived from miR-146-expressing MSCs significantly reduced glioma xenograft growth [67]. Identical to GBM, hepatocellular carcinoma (HCC) is also highly resistant to conventional chemotherapy. Considering that microRNA-122 is a crucial promoter of the chemical sensitivity of HCC cells, Lou et al. [18] aimed to determine whether adipose tissue-derived MSC (AMSC) exosomes can be used for miR-122 delivery. The results indicated that miR-122 exported from AMSC-derived exosomes enhances the chemical sensitivity of HCC. Similarly, miR-targeting therapies have also been applied to treat other types of tumors. As demonstrated by Shimbo et al. [68], the delivery of synthetic miR-143 formed by MSC-derived exosomes significantly reduces the migration of osteosarcoma cells. Che et al. also focused on miR-143 and investigated the effect of hBMSC-derived exosomal miR-143 on prostate cancer; they found that miR-143 negatively targets TFF3 to suppress cancer progression [69]. Synthetic small stem RNA (siRNA), which can be used to selectively inhibit a target gene, has great potential in cancer treatment. MSC-derived exosomes can be used as delivery vehicles for synthetic siRNA. In the study by Greco et al. [70], treating bladder cancer cells with exosomes electroporated with PLK-1 siRNA successfully knocked down PLK-1 mRNA and protein, resulting in apoptosis and necrosis of bladder cancer cells. Kalimuthu et al. observed that MSC exosomes can be used as drug delivery vehicles, and thus, they could deliver PTX to breast cancer cells [71]. Moreover, Altanerova et al. [72] found that MSC-derived iron oxide exosomes can be combined with magnetotherapy for target resection of tumor cells.

## 5. The Role of Tumor Cell-Derived Exosomes on MSCs

Extensive research confirms that tumor-derived exosomes contain molecular and genetic signals that are able to induce modifications in MSCs and transform them from normal nutrition to tumorigenic, which produce factors necessary for tumor growth [73, 74]. Lindoso et al. reported, for example, that renal cancer stem cell-derived exosomes promote MSC migration to the tumor and induce expression of the tumorigenic phenotype in these MSCs [75]. Another study has shown that exosomes produced by primary or metastatic colorectal cancer can reprogram mesenchymal stem cells, inducing morphological and functional changes that are beneficial to tumor growth and metastasis [76]. Exosomes from prostate or breast cancer cells can induce bone marrow mesenchymal stem cell differentiation into myeloid fibroblasts that overexpress alpha smooth muscle actin [77]. Moreover, exosomes derived from AML cells can transform MSCs into leukemia growth-permitting cells and inhibit normal hematopoiesis in vivo [78]. Yeon et al. proved melanoma-derived exosomes trigger endothelial to mesenchymal transition followed by the induction of cancer-associated fibroblasts [79]. The overall conclusion of these experiments is that MSCs reprogrammed by tumor-derived exosomes are essential to tumor progression.

## 6. Discussion

Researchers have discovered that MSC-derived exosomes may play two roles in the tumor microenvironment [80], but few reports have addressed how and why this is the case. The complex cellular and molecular interactions between MSCs and the surrounding tissue microenvironment may lead to different results. MSCs can migrate to tumors and evolve into diverse types of cells, such as TA-MSCs and CAFs [15]. Cancer-derived signals can regulate the phenotype of cancer-recruited MSCs, making them a part of the cancer mass; these cancer-recruited MSCs possess characteristics that are distinct from other tissue-derived MSCs or BM-MSCs [81]. Typically, the difference between noncancer-related MSCs and CAFs may be accounted for by the responses of cytokines and exosomes produced in the tumor microenvironment [82]. In addition, MSCs are more frequently detected in cancer tissues than in adjacent normal tissues, which have a greater proliferation capacity [83]. Depending on the source, exosomes exhibit distinct characteristics and secretory factors, which may be related to their biogenesis and targeting and putative immune functions [84]. This indicates the necessity of assessing the source of exosomes formed by the microenvironment. Moreover, exosome-secreting factors represent the roles of exosomes in establishing and altering the tumor microenvironment. In general, the effects of MSC-derived exosomes in promoting or inhibiting tumors and cancers seem to depend on the source of MSC-derived exosomes; depending on the tumor or cancer microenvironment, MSCs may be transformed into CAFs or TA-MSCs. As suggested in some studies, glioma-associated human MSC-derived exosomes enhance the aggressiveness of glioma [35]. However, the exosomes derived from BM-MSCs carry antitumor miRNAs, which significantly reduce the growth of the glioma xenograft [67]. Coincidentally, exosomes released by human breast cancer AMSCs induce breast cell proliferation and migration [36–51]. Exosomes derived from TA-MSCs accelerate breast cancer progression [44], but hBMSC-derived exosomes have been confirmed to stimulate cycling quiescence and breast cancer dormancy after metastasizing to the bone marrow [59, 60]. Roccaro et al. [38] noted that BM-MSC-derived exosomes in patients with multiple myeloma supported the progression of multiple myeloma cells, whereas exosomes isolated from normal hBMSCs might metastasize to the low miR-15a level, thus inhibiting the development of multiple myeloma cells. In several studies, tumor growth inhibition has been observed when MSC-derived exosomes are introduced into the established tumors or the cultured tumor cells. By contrast, numerous studies have demonstrated that MSC-derived exosomes promote tumor growth when the MSCs are cocultured with tumor cells. Exosomes isolated from a coculture of MSCs/breast cancer cells were found to enhance the growth of cancer cells [41]. Meanwhile, exosomes prepared from the supernatant of BM-MSCs were demonstrated to suppress the in vitro angiogenesis of breast cancer cells [57]. Moreover, Karaoz et al. [85] reported that the proliferation rate of cancer cells significantly increased when they were cocultured with WJ-MSCs but not when they were cocultured with cancer cells treated with pure MSC-derived exosomes.

**Figure 4:**
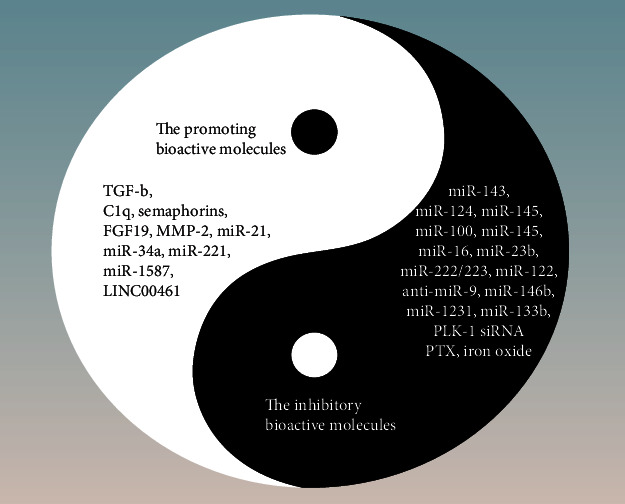
MSC-derived exosomes shuttle various bioactive molecules to inhibit or promote tumor growth. Black areas are inhibitory bioactive molecules, and white areas are promotional bioactive molecules.

The bioactive molecules shuttled by MSC-derived exosomes reprogram recipient cells, and the contents of MSC-derived exosomes have been determined to include protein, microRNA, lncRNA, liposomes, and other molecules. Thus, another mechanism related to the two-sided effects of MSC-derived exosomes on tumors may be related to the differences in the components contained in MSC-derived exosomes. As shown in Figure 4, for example, MSC-derived exosomes play a role in transporting MMP-2 [41], miR-21, and miR-34a [36], which have been demonstrated to be involved in cancer cell survival [86] and proliferation [2, 87], and MMP-2, which has also been demonstrated to be overexpressed in breast cancer cells; additionally, the high expression of MMP-2 is associated with poor prognosis [88, 89]. Although MSC-derived exosomes can inhibit cancer cells by transporting miR-100 and miR-16, among others, miR-100 induction could counteract the tumor-promoting effect of EMT-induced transcription factors [90], and miR-16 was demonstrated to downregulate VEGF expression [91]. Moreover, TGF-b, C1q, and semaphorins have been found to be involved with MSC-derived exosomes in promoting breast cancer development [44]; however, miR-23b [59] and miR-222/223 [60] were found to be transported by MSC-derived exosomes to suppress breast cancer development. Similarly, MSC-derived exosomes that transport miR-1587 [35] increase the proliferation and self-renewal of GSCs, whereas shuttled miR-146b [67] inhibits glioma growth. In general, MSC-derived exosomes transport different cargoes that may have different effects on tumors.

Finally, the effects of MSC-derived exosomes may vary depending on the tumor type or stage of tumor development. For example, many studies have revealed that MSC-derived exosomes facilitate gastric cancer growth and migration (Table 1) and that exosomes derived from gastric cancer cells stimulate CAF differentiation of MSCs [92]. However, MSC-derived exosomes appear to only exhibit an inhibitory effect on prostate cancer, as indicated in Table 2. MSC-derived exosomes can inhibit cancer cell migration and invasion [69] or suppress cancer progression [54]. According to relevant research, MSC-derived exosomes are more inclined to inhibit angiogenesis and metastasis in breast cancer [57] and stimulate cycling quiescence and dormancy of cancer cells, preventing metastasis [59, 60]. By contrast, for HCC [18] and GBM [66], MSC-derived exosomes are more likely to affect chemoresistance.

## 7. Conclusion

As a newly discovered intercell information transmission tool, MSC-derived exosomes play crucial roles in tumor development and are a major focus of recent research. The regulatory effect of MSC-derived exosomes on tumors remains debated and has three potential mechanisms. First, the source of MSC-derived exosomes may be influential; MSCs may undergo transformation in the tumor microenvironment or cancer microenvironment, and CAF- and TA-MSC-derived exosomes promote tumor development, whereas MSC-derived exosomes from healthy tissue could inhibit tumor growth. Second, another mechanism may be related to the differences in the components contained in MSC-derived exosomes. Finally, MSC-derived exosomes have different effects on different tumors. Of particular note, MSC-derived exosomes are ideal carriers for tumor-targeted therapy and display several unique advantages, including simple acquisition, easy access to recipient cells, and strong plasticity. Consequently, thorough research is necessary for the rapid advancement of MSC-derived exosome treatments.

## Figures and Tables

**Table 1:** Tumor/cancer-promoting cargoes transported by MSC-derived exosomes.

Cargo type	Source of exosome	Target cancer	Outcome	Exosomal cargo/pathway	Reference
Pathway	Human umbilical cord MSCs	Gastric cancer cells	Promotion of cell growth and migration	Activated Akt pathway	[47]
	Human bone marrow MSCs	Gastric cancer cells	Promotion of tumor growth	Activated kinase1/2 (ERK1/2) pathway	[50]
	Human bone marrow MSCs	Osteosarcoma and gastric cancer cells	Promotion of tumor growth	Activated Hedgehog pathway	[49]
	Human breast cancer AMSCs	Breast cancer cells	Promotion of cell growth	Activated Hippo signaling pathway	[51]
	Human MSCs	Gastric cancer cells	Conferral of drug resistance	Activated CaM-Ks and Raf/MEK/ERK pathways	[53]
	Human adipose-derived MSCs	Breast cancer cells	Promotion of cell migration	Activated Wnt pathway	[52]

Protein	Human and mouse tumor-educated MSCs	Breast cancer cells	Acceleration of cancer progression	TGF-b, C1q, and semaphorins	[44]
	MSCs	Nasopharyngeal carcinoma cells	Facilitation of tumor progression	FGF19	[43]
	MSCs	Breast cancer cells	Support of cell reorganization and growth	MMP-2	[41]

miRNA	SD human MSCs	Breast cancer cells	Support of cancer cell proliferation and metastasis	miR-21 and 34a	[36]
	Gastric cancer tissue-derived MSCs	Gastric cancer cells	Promotion of cell proliferation and migration	miR-221	[37]
	Glioma-associated MSCs	Glioblastoma cells	Enhancement of aggressiveness	miR-1587	[35]

lncRNA	MSCs	Multiple myeloma cells	Promotion of proliferation and suppression of apoptosis	LINC00461	[39]

**Table 2:** Tumor/cancer-inhibiting cargoes transported by MSC-derived exosomes.

Cargo type	Source of exosome	Target cancer/cells	Outcome	Exosomal cargo/pathway	Reference
miRNA	Human bone marrow MSCs	Osteosarcoma cells	Inhibition of tumor cell migration	miR-143	[68]
	MSCs	Glioma cells and glioma stem cells	Reduction of cell migration and self-renewal	miR-124 and miR-145	[55]
	Human MSCs	Breast cancer cells	Suppression of angiogenesis	miR-100	[57]
	MSCs	Prostate cancer cells	Suppression of cancer progression	miR-145	[54]
	MSCs	Breast cancer cells	Suppression of angiogenesis	miR-16	[58]
	Human bone marrow MSCs	Breast cancer cells	Promotion of dormancy	miR-23b	[59]
	MSCs	Breast cancer cells	Stimulation of cycling quiescence and dormancy	miR-222/223	[60]
	Human bone marrow MSCs	Prostate cancer cells	Inhibition of cell migration and invasion	miR-143	[69]
	Human adipose-derived MSCs	Hepatocellular carcinoma	Cancer cells rendered sensitive to chemotherapeutic agents	miR-122	[18]
	Normal bone marrow MSCs	Multiple myeloma	Inhibition of tumor promotion	—	[38]
	MSCs	Glioblastoma multiforme cells	Reversal of chemoresistance	Anti-miR-9	[66]
	MSCs	Glioma	Reduction of progression and metastasis	miR-146b	[67]
	MSCs	Pancreatic cancer cells	Inhibition of cancer activity	miR-1231	[61]
	MSCs	Glioma	Suppression of progression	miR-133b	[56]

siRNA	MSCs	Bladder cancer cells	Induction of apoptosis and necrosis	PLK-1 siRNA	[70]

Pathway	MSCs	Human leukemia cells	Enhancement of apoptosis	Activated caspase pathway	[62]

Drug	MSCs	Breast cancer cells	Reduction in viability	PTX	[71]
	Human MSCs	HeLa cells	Ablation of cancer cells	Iron oxide	[72]

## Abbreviations

CAFs: Cancer-associated fibroblasts
MSCs: Mesenchymal stem cells
TA-MSCs: Tumor-associated mesenchymal stem cells
MVBs: Multivesicular bodies
ESCRT: Endosomal sorting complex required for transport
hMSCs: Human mesenchymal stem cells
AMSCs: Adipose tissue-derived mesenchymal stem cells
WJ-MSCs: Wharton's jelly mesenchymal stem cells
BM-MSCs: Bone marrow-derived mesenchymal stem cells
MM: Multiple myeloma
NPC: Nasopharyngeal carcinoma cells
EMT: Epithelial-mesenchymal transition
TMZ: Temozolomide
PTX: Paclitaxel.
